# Investigation of germline variants in Bahraini women with breast cancer using next-generation sequencing based-multigene panel

**DOI:** 10.1371/journal.pone.0291015

**Published:** 2023-09-01

**Authors:** Ghada Al-Kafaji, Ghufran Jassim, Amani AlHajeri, Amna Mohamed Tayeb Alawadhi, Mariam Fida, Ibrahim Sahin, Faisal Alali, Elias Fadel

**Affiliations:** 1 Department of Molecular Medicine and Al-Jawhara Centre for Molecular Medicine, Genetics, and Inherited Disorders, College of Medicine and Medical Sciences, Arabian Gulf University, Manama, Kingdom of Bahrain; 2 Department of Family Medicine, Royal College of Surgeons in Ireland-Bahrain, Manama, Kingdom of Bahrain; 3 Department of Genetics, Salmaniya Medical Complex, Manama, Kingdom of Bahrain; 4 Bahrain Oncology Center, King Hamad University Hospital, Manama, Kingdom of Bahrain; 5 North western Hospital, Chicago Medical School, North Chicago, Illinois, United States of America; The University of Queensland Faculty of Medicine, AUSTRALIA

## Abstract

Germline variants in *BRCA1* and *BRCA2* (*BRCA1/*2) genes are the most common cause of hereditary breast cancer. However, a significant number of cases are not linked to these two genes and additional high-, moderate- and low-penetrance genes have been identified in breast cancer. The advent of next-generation sequencing (NGS) allowed simultaneous sequencing of multiple cancer-susceptibility genes and prompted research in this field. So far, cancer-predisposition genes other than *BRCA1*/*2* have not been studied in the population of Bahrain. We performed a targeted NGS using a multi-panel covering 180 genes associated with cancer predisposition to investigate the spectrum and frequency of germline variants in 54 women with a positive personal and/or family history of breast cancer. Sequencing analysis revealed germline variants in 29 (53.7%) patients. Five pathogenic/likely pathogenic variants in four DNA repair pathway-related genes were identified in five unrelated patients (9.3%). Two *BRCA1* variants, namely the missense variant c.287A>G (p.Asp96Gly) and the truncating variant c.1066C>T (p.Gln356Ter), were detected in two patients (3.7%). Three variants in non-*BRCA1/2* genes were detected in three patients (1.85% each) with a strong family history of breast cancer. These included a monoallelic missense variant c.1187G>A (p.Gly396Asp) in *MUTYH* gene, and two truncating variants namely c.3343C>T (p.Arg1115Ter) in *MLH3* gene and c.1826G>A (p.Trp609Ter) in *PMS1* gene. Other variants of uncertain significance (VUS) were also detected, and some of them were found together with the deleterious variants. In this first application of NGS-based multigene testing in Bahraini women with breast cancer, we show that multigene testing can yield additional genomic information on low-penetrance genes, although the clinical significance of these genes has not been fully appreciated yet. Our findings also provide valuable epidemiological information for future studies and highlight the importance of genetic testing, and an NGS-based multigene analysis may be applied supplementary to traditional genetic counseling.

## Introduction

Breast cancer is the most common malignancy and the leading cause of cancer death among women globally [1]. The incidence of breast cancer is significantly high in the Western world with an estimated lifetime risk of one in nine [2]. The prevalence of breast cancer is also increased in Arab and Middle-Eastern women, with an earlier diagnosis age compared to the Western countries [3]. The Gulf Cooperation Council (GCC) is comprised of Bahrain, Saudi Arabia, United Arab Emirates (UAE), Qatar and Oman. Bahrain (officially the Kingdom of Bahrain) is an island nation located in a bay on the southwestern coast of the Arabian Gulf. It is the smallest among the GCC states and rich in its culture and history. According to 2021 statistics, the total number of population in Bahrain is 1,504,365, of whom 719,333 (47.8%) are Bahraini and 785,032 (52.2%) are non-Bahraini. The majority of non-Bahraini population are form the major part of the working force [4]. The Arabs represent a mix of many ethnicities with a distinctive genetic profile. Genome sequencing analysis of 104 unrelated natives of the Arabian Peninsula placed indigenous Arabs as direct descendants of the first Eurasian populations established by the out-of-Africa migrations [5]. A study by Bahri et al., [6] showed that Bahrainis ancestors were mainly emigrants from Arabia and Iran according to Alu insertion polymorphism analysis. Another study by Garcia-Bertrand et al., [7] showed that UAE and Bahrain share 23.7% and 22.9%, respectively, of their DNA with Southwest Asian populations. Some of the factors that contribute to the unique genetic makeup in the GCC countries include consanguinity, which is very high among the Arabs [8], and waves of migration of different ancestral populations into the region. Among the GCC states, Bahrain has a high incidence of breast cancer [9, 10], with an overall mean age at diagnosis of 50.9 years [9]. Although, there is a lack of studies related to genetic epidemiology of breast cancer in Bahrain, the recent foundation of Bahrain genome project [11] will help in the identification of specific genetic variants associated with susceptibility to diseases including breast cancer, and will contribute to the development of diagnostic methods and personalized approaches for disease management.

While the majority of breast cancer cases are sporadic, about 5–10% of cases are hereditary and 15–20% show familial aggregation [12, 13]. Germline variants in two high penetrance genes, *BRCA1* and *BRCA2 (BRCA1/2)*, are the most common cause of hereditary breast cancer [14–17]. The cumulative risk of breast cancer in carriers of variants in *BRCA1* and *BRCA2 genes* was reported to be 72% and 69%, respectively [18], and women with *BRCA1/2* variants are advised to undergo prophylactic risk-reducing surgery to decrease cancer-related mortality [19]. Remarkably, *BRCA1/2* germline variants have been regarded for some target therapies such as Poly (ADP-ribose) polymerase (PARP) inhibitors to improve survival, especially in patients with early or metastatic breast cancer [20–23]. Nevertheless, about 30% of the breast cancer patients who have a family history of inherited breast cancer do not carry *BRCA1*/*2* variants [24–28]. Moreover, approximately a four-fold risk of breast cancer was reported in women with a significant family history of breast cancer but who tested negative for *BRCA1* or *BRCA2* variants [26]. Other high-penetrance genes such as *CDH1*, *PALB2*, *PTEN* and *TP53* and additional moderate- and low-penetrance genes may also increase breast cancer risk [27, 28]. Many of these genes are involved in DNA damage response, homologous recombination (HR) repair, or mismatch repair pathways [27, 28]. The National Comprehensive Cancer Network (NCCN) guidelines for Genetic/Familial High- Risk Assessment: Breast, Ovarian, and Pancreatic (version 2.2022, accessed on March 2022) recommend genetic testing for additional genes beside *BRCA1/2* genes [29]. Therefore, screening for breast cancer susceptibility genes provides an essential insight into the contribution of clinically relevant variants, which can impact breast cancer management for patients and at-risk family members.

The advent of next-generation sequencing (NGS) has greatly expanded the use of multigene panel testing. The application of NGS-based-multigene testing, which enables sequencing of a large number of genes simultaneously, can provide more information than a single-gene test and has a great implication in breast cancer risk prediction and selection of precise treatment.

More than 200 multigene panels in which *BRCA1*/2 genes are included have been proposed by academic or commercial laboratories [30]. In this context, several recent studies have used multigene panels and provided valuable information of the possible role of several genes in hereditary breast cancer risk. For instance, Yang et al., [31] investigated germline variants in a panel containing 152 genes associated with cancer in a cohort of Chinese breast cancer patients, and found variants in genes other than *BRCA1/2* such as *TP53* and *CDH1*, as well as DNA mismatch repair genes and Fanconi anemia genes. Shin et al., [32] analysed germline variants in a panel containing 64 cancer predisposition genes in Korean breast cancer patients with clinical features of hereditary cancer syndrome, and detected *BRCA1/2* variants in 63.2% of patients were carriers for and 40.0% were carriers for non-*BRCA1/2* genes such as *CHEK2*, *PALB2*, *MRE11*, and *RAD50*. Using a panel containing 93 cancer predisposition genes in Egyptian patients with familial breast cancer, Nassar et al., [33] identified 27 deleterious germline variants in 11 cancer susceptibility genes including *ATM*, *BRCA1*, *BRCA2*, *VHL*, *MSH6*, *APC*, *CHEK2*, *MSH2*, *MEN1*, *PALB2*, and *MUTYH*. These studies showed differences in the spectrum and prevalence of germline variants in breast cancer patients among ethnicities, signifying the need for respective studies in ethnically specific populations. The identification of population-specific variants is crucial to incorporate accurate genetic testing into clinical practice to meet the need for more specific precision therapy. To date, only one study in Bahrain was conducted to investigate the frequency of germline variants in familial breast cancer women, and it was solely focused on *BRCA1/2* gene profiling [34]. However, cancer-predisposition genes other than *BRCA1*/*2* have not been well studied in the population of Bahrain. The aim of this study was to investigate the spectrum and frequency of germline variants in Bahraini women with a positive personal and/or family history of breast cancer using a targeted NGS-multi-panel covering 180 genes associated with cancer predisposition.

## Methods

### Patients

Women diagnosed with breast cancer in the period between 1^st^ January 2019 and 31^st^ December 2020, and reported to the Bahrain National Tumor Board at the Bahrain Oncology Center (BOC)—King Hamad University Hospital (KHUH) in Bahrain were recruited in this study. The period was chosen because initial search revealed that recording and notification of breast cancer cases were at maximum during that period. Patients included in this study met one of the following criteria based on the latest National Comprehensive Cancer Network (NCCN) guidelines for Genetic/Familial High- Risk Assessment: Breast, Ovarian, and Pancreatic (version 2.2022, accessed on March 2022) https://www.nccn.org/guidelines/guidelines-detail?category=2&id=1503 for genetic testing: 1) Personal history of breast cancer at age ≤ 50 years; 2) personal history of breast cancer at any age with treatment indications (e.g. PARP inhibitors for metastatic breast cancer) or triple-negative breast cancer; 3) personal history of breast cancer at any age with ≥ 1 close relative with breast cancer at age ≤ 50 years, or with other cancers (ovarian, pancreatic and prostate cancers); 4) personal history of breast cancer at any age with ≥ 2 close blood relative with either breast cancer or prostate cancer (any grade). Genetic counseling was offered to the patients in the Medical Genetics clinic at KHUH to assess their eligibility for inclusion and to explain the purpose of genetic testing. At this time, the personal and family history data and pedigrees of patients were documented. All patients were not related by means of three-generation pedigree cross-comparison to each other, and only one patient per family was included. Electronic medical records of patients were reviewed for clinical information including age at diagnosis, laterality of breast cancer, histology type and grade. The expression status of estrogen receptor (ER), progesterone receptor (PR), and Her2 was also obtained from the oncology reports of patients. The molecular subtypes of breast cancer were divided into the following categories: Luminal A (ER+, PR+, HER2-), Luminal B (ER+, PR-, HER2+), HER2+ markers and triple-negative (TNBC). All data were collected and accessed for research purposes between 15^th^ May 2022 and 26^th^ June 2022.

### Ethical consideration

The study was conducted in accordance with the Helsinki declaration. Informed written consents were obtained from all patients. Ethical approval was obtained from the Institutional Review Board at King Hamad University Hospital (KHUH) (Reference # 22–509).

### Genomic DNA extraction

Genomic DNA was extracted from EDTA peripheral blood samples using the MagNa Pure LC DNA Isolation Kit (Roche Diagnostics GmbH, Mannheim), according to the manufacturer’s recommended protocol. DNA concentration was evaluated by the Nano Drop^™^ 2000 spectrophotometer (Thermos Fisher, Yokohama, Japan) and DNA quality was assessed using 0.8% agarose gel electrophoresis.

### Gene selection

A targeted gene-panel covering 180 cancer susceptibility genes was chosen in this study. The genes were selected based on the NCCN guidelines and published studies in PubMed with a keyword search of "Neoplasms” AND “germline mutation”, with the possibility to identify novel candidate genes associated to hereditary breast cancer risk. The genes include: *ALK*, *ANKRD26*, *AP2S1*, *APC*, *AR*, *ATM*, *ATR*, *AXIN2*, *BAP1*, *BARD1*, *BLM*, *BMPR1A*, *BRAF*, *BRCA1*, *BRCA2*, *BRIP1*, *BUB1B*, *CBL*, *CD70*, *CD82*, *CD73*, *CDH1*, *CDH10*, *CDK4*, *CDKN1A*, *CDKN1B*, *CGKN1C*, *CDKN2A*, *CDKN2B*, *CDKN2C*, *CEBPA*, *CEP57*, *CHEK2*, *CRTAC1*, *CTNNA1*, *CTRC*, *CYLD*, *DDB2*, *DDX41*, *DICER1*, *DIS3L2*, *DKC1*, *EFNA1*, *EGFR*, *ELAC2*, *EPCAM*, *ERCC1*, *ERCC2*, *ERCC3*, *ERCC4*, *ERCC5*, *ETV6*, *EXO1*, *EXT2*, *FAM175A*, *FANCA*, *FANCB*, *FANCC*, *FANCD2*, *FANCE*, *FANCF*, *FANCG*, *FANCI*, *FANCM*, *FAT1*, *FEN1*, *FH*, *FLCN*, *GALNT12*, *GREM1*, *HNF1A*, *HNF1B*, *HOXB13*, *HRAS*, *IKZF1*, *KDR*, *KIF1B*, *KIT*, *KITLG*, *KRAS*, *LMO1*, *LZTR1*, *MAP2K1*, *MAP2K2*, *MAX*, *MEN1*, *MET*, *MITF*, *MLH1*, *MLH3*, *MPL*, *MRE11A*, *MSH2*, *MSH3*, *MSH6*, *MSR1*, *MUTYH*, *NBN*, *NF1*, *NF2*, *NRAS*, *NSD1*, *NSUN2*, *NTHL1*, *NTRK1*, *NTRK2*, *NTRK3*, *PALB2*, *PALLD*, *PAX5*, *PDGFRA*, *PMS1*, *PMS2*, *POLD1*, *POLE*, *POLH*, *POLQ*, *POT1*, *PPM1D*, *PRF1*, *PRKAR1A*, *PRSS1*, *PTCH1*, *PTEN*, *PTPN11*, *PTPN13*, *RAD50*, *RAD51C*, *RAD51D*, *RAD54L*, *RAF1*, *RASA2*, *RB1*, *RECQL*, *RECQL4*, *REST*, *RET*, *RHBDF2*, *RIT1*, *RANASEL*, *RNF43*, *RPS20*, *RRAS*, *RUNX1*, *SAMD9*, *SBDS*, *SDHA*, *SDHAF2*, *SDHB*, *SDHD*, *SETBP1*, *SHOC2*, *SLX4*, *SMAD4*, *SMARCA4*, *SMARCB1*, *SMARCE1*, *SOS1*, *SOS2*, *SPINK1*, *SPOP*, *SPRED1*, *SRP72*, *STK11*, *SUFU*, *TERT*, *TGFBR2*, *TINF2*, *TMEM127*, *TP53*, *TP63*, *TSC1*, *TSC2*, *VHL*, *WRN*, *WT1*, *XPA*, *XPC*, *XRCC2*, *XRCC3*.

### Next generation sequencing and data analysis

**Fig 1:**
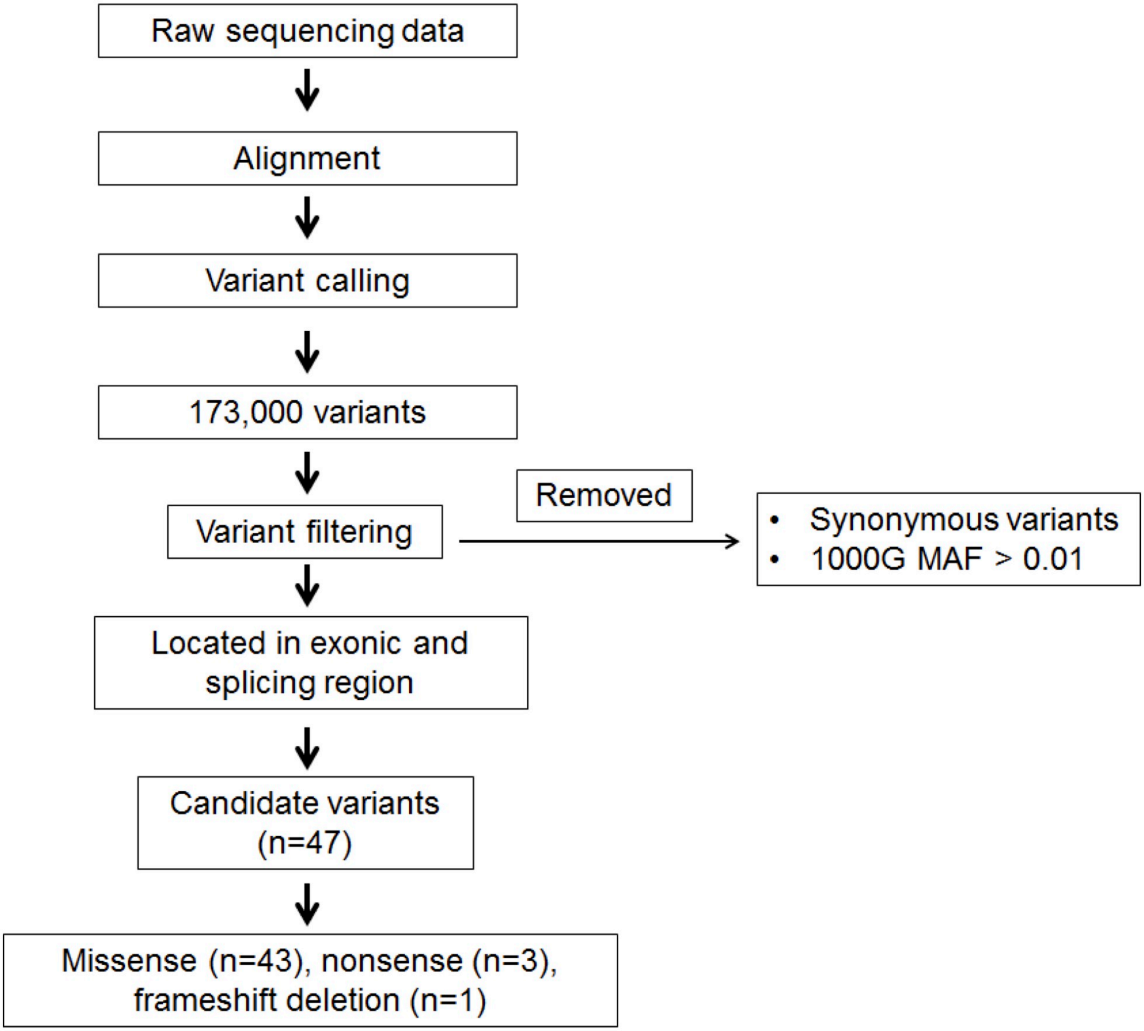
Summary of the data analysis pipeline in this study.

The targeted region included all coding exons and exon–intron boundaries of each gene included in the panel. Library preparation was performed using the Ion AmpliSeqTM Exome RDY Kit (Thermo Fisher Scientific, Inc.) according to the manufacturer’s instruction. This kit enables high-efficiency enrichment with target base coverage of 94.47% at depth ≥ 20X and quality threshold of 97.71%. The libraries were sequenced on the Ion ProtonTM following the manufacturer’s instructions (Thermo Fisher Scientific, Inc.). After filtering, reads were aligned against the human reference genome hg19/GRCh37 using the Torrent Mapping Alignment Program v.5.0.13 included in the Torrent Suite software for Sequencing Data Analysis V.5.0.4 (Thermo Fisher Scientific, Inc.). Variant calling was performed using a computational pipeline built on the variant caller Platypus v0.81. For final variant calling, filtering was carried out to eliminate erroneous base calling by visually examining variants using Integrative Genomics Viewer software (http://www.broadinstitute.org/igv). Candidate variants were obtained after filtering by gene function, focusing on those genes with a potential role in cancer. Nonsynonymous (missense and nonsense); insertions and deletions (InDels); and splicing variants, and variants occur in the 1000 Genome database with a minor allele frequency of < 0.01 were included. Synonymous variants and variants occur in the 1000 Genome database with a minor allele frequency of > 0.01 were excluded [35]. Summary of the data analysis pipeline in the present study is shown in Fig 1. For interpretation of variants, we followed the American College of Medical Genetics and Genomics (ACMG) 2015 guidelines [36], in which variants were classified into five categories: Pathogenic, likely pathogenic (> 90% certainty of a variant being disease-causing), variant of uncertain significance (VUS), likely benign (> 90% certainty of a variant being benign) and benign. Accordingly, variants were classified as pathogenic with very strong evidence of pathogenicity (PVS1) if they produced premature termination codons associated with non-functional or truncated proteins including nonsense, frameshift and splice-site mutations. Variants that do not fulfil the criteria for pathogenic/likely pathogenic, benign/likely benign or with conflicting interpretation of pathogenicity were classified as variants of uncertain significance (VUS).

### Bioinformatics analysis

Unclassified variants were analyzed using *in silico* prediction tools that use sequence homology, evolutionary conservation, and protein structural information [37]. These tools include: 1) Combined Annotation Dependent Depletion (CADD) integrates multiple information sources including conservation, structure-based features and functional information, and categorizes variants as benign or deleterious using a machine learning approach. CADD predicts a continuous PHRED and a cut-off score above 15 is considered deleterious. 2) REVEL is an ensemble method for predicting the pathogenicity of variants based on a combination of scores from 13 tools including MutPred, FATHMM, VEST, PolyPhen, SIFT, PROVEAN, MutationAssessor, MutationTaster, LRT, GERP, SiPhy, phyloP, and phastCons. REVEL cut-off score above 0.5 is considered deleterious.

## Results

### Characteristics of patients

The characteristics of 54 women with breast cancer included in this study are summarized in Table 1. The mean age at diagnosis of breast cancer was 48.7 years (± 9.5), ranging from 27 to 65 years. Three patients (5.6%) were diagnosed before the age of 30 years, 18 patients (33.3%) were diagnosed at age ≤ 40 years, 16 patients (29.6%) were diagnosed at age ≤ 50 years, 15 patients (27.7%) were diagnosed at age ≤ 60, and 2 patients (3.7%) were diagnosed at age > 60 years. The majority of patients (53, 98.1%) were diagnosed with unilateral breast cancer and only one patient was diagnosed with primary bilateral breast cancer. Most breast cancers were invasive ductal carcinoma (45, 83.3%), and ductal carcinoma in situ was reported in 7 patients (13%). Regarding the molecular subtype, 30 (55.6%) were Luminal A, five (9.3%) Luminal B, five (9.3%) HER2+, five (9.3%) HER2/ER+, and five (9.3%) TNBC. One patient was diagnosed with thyroid cancer alongside breast cancer. Of all breast cancer patients, 44 (81.5%) had at least one first- and/or second-degree blood relative with breast cancer, and two (3.7%) had a family history of ovarian cancer. Furthermore, 40 patients (74.1%) had a family history of other cancers (such as colon, colorectal, endometrial, testicular, gastric, pancreatic, prostate, leukemia).

**Table 1:** Characteristics of patients.

Characteristics of patients (n = 54)	no (%)
Age at diagnosis (years)	
< 30	3 (5.6)
30–40	18 (33.3)
41–50	16 (29.6)
51–60 years	15 (27.7)
> 60 years	2 (3.7)
Laterality	
Unilateral	53 (98.1)
Bilateral	1 (1.9)
Histology of breast tumor	
Invasive carcinoma	45 (83.3)
In situ carcinoma	7 (13)
Unknown	2 (3.7)
Molecular subtypes of breast tumor	
Luminal A	30 (55.6)
Luminal B	5 (9.3)
HER2 positive	5 (9.3)
HER2/ER positive	5 (9.3)
TNBC	5 (9.3)
Unknown	4 (7.4)
Breast cancer patients with personal history of another type of cancer	1 (1.9)
*Family history of breast cancer	
Yes	44 (81.5)
No	10 (18.5)
Family history of ovarian cancer	
Yes	2 (3.7)
No	52 (96.3)
Family history of other cancers	
Yes	40 (74.1)
No	14 (25.9)

Data are presented as number and percentage (%).

*Family history of breast cancer in the first- and/or second-degree blood relatives of the patients.

### Gene-panel findings

**Fig 2:**
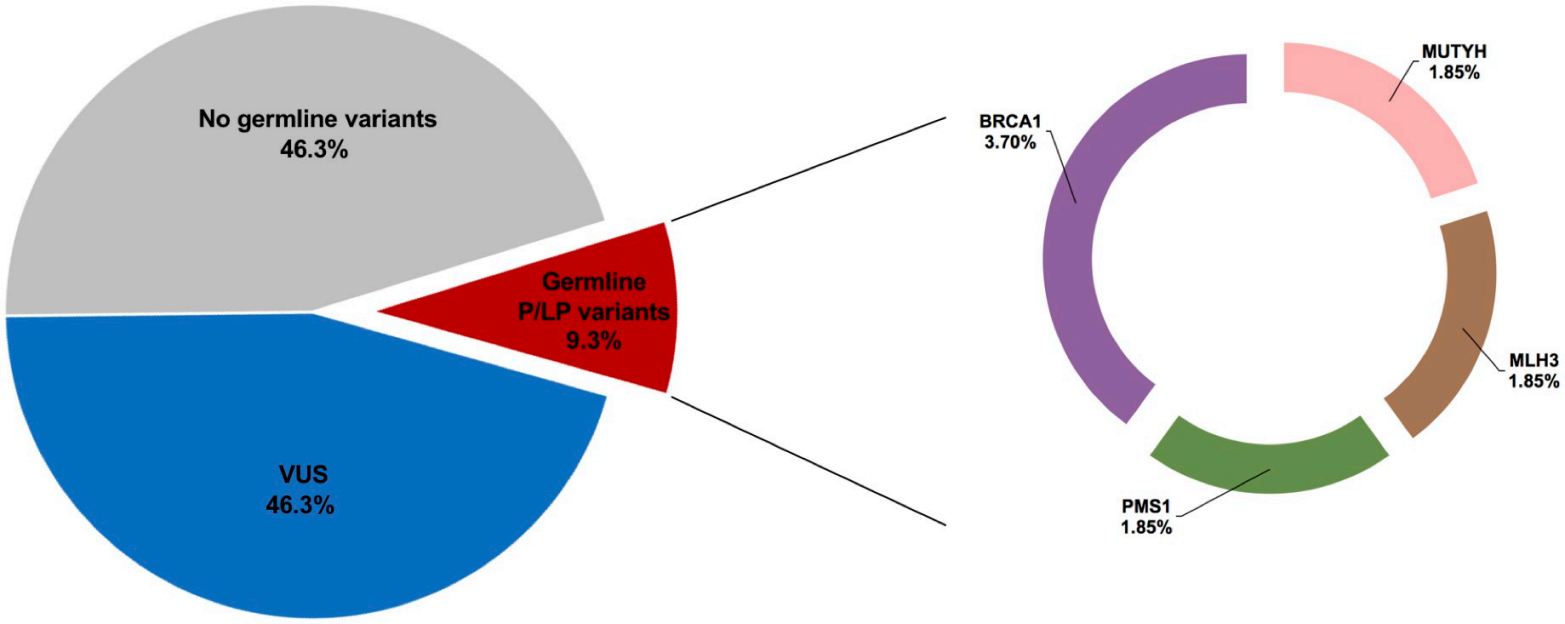
Gene-panel testing for 54 breast cancer patients. A) Cases where pathogenic/likely pathogenic variants were identified. B) Percentage of pathogenic/likely pathogenic variants identified in each gene.

Sequencing data of 54 breast cancer patients in multi-gene panel testing showed that 25 (46.3%) patients were not carriers for germline variants and 29 (53.7%) patients were carriers for germline variants. The results revealed 173,000 distinctive variants before filtering. After variant filtering a total of 47 germline variants in 33 cancer susceptibility genes were identified, including 43 missense variants, three nonsense variants, and one frameshift deletion. Five patients (9.3%) were pathogenic or likely pathogenic variant carriers. Two patients (3.7%) were identified as harboring *BRCA1* gene variants. Three other patients were carriers for non-*BRCA1/2* variants including one in *MUTYH* gene (1.85%), one in *MLH3* gene (1.85%) and one in *PMS1* gene variant (1.85%). Twenty five patients (46.3%) were found to carry VUS, since some of them also carried pathogenic or likely pathogenic variants. The identified genes with germline variants and their association with various cancer types and syndromes are listed in Table 2. Summary of gene-panel findings is provided in Fig 2.

**Table 2:** List of identified genes with germline variants in this study and their association with various cancer types and syndromes.

Gene	Chromosome	RefSeq	Associated syndromes
*APC*	5	NM_000038.5	Familiar Adenomatous Polyposis (FAP)
*ATM*	11	NM_000051.3	Ataxia-Telangiectasia (recessive)
*BRCA1*	17	NM_007294.2	Hereditary Breast and Ovarian Cancer Syndrome (HBOC)
*CBL*	11	NM_005188.3	Noonan syndrome-like disorder, Jacobsen syndrome, many cancers including acute myeloid leukemia
*CHEK2*	22	NM_007194.3	Sarcomas, breast cancer, and brain tumors
*CTRC*	1	NM_007272.2	Pancreatitis, hereditary and Prss1-hereditary pancreatitis
*ERCC3*	2	NM_000122.1	Xeroderma pigmentosum B, Cockayne’s syndrome, trichothiodystrophy
*ERCC4*	16	NM_005236.2	Xeroderma pigmentosum complementation group F (XP-F), or xeroderma pigmentosum VI (XP6)
*FAM175A (ABRAXAS1*)	4	NM_139076.2	Inherited cancer-predisposing syndrome, Bap1 tumor predisposing syndrome
*FANCI*	15	NM_001113378.1	Fanconi anemia complementation group I and group A
*FANCM*	14	NM_020937.3	Fanconi anemia, premature ovarian failure, and spermatogenic failure
*FAT1*	4	NM_005245.3	Many cancers (e.g., head and neck squamous cell carcinoma)
*FLCN*	17	NM_144997.5	Birt-Hogg-Dube syndrome
*KIF1B*	1	NM_015074.3	Charcot-Marie-Tooth Disease Type 2A1, Neuroblastoma
*MLH3*	14	NM_001040108.1	Lynch syndrome
*MSH2*	2	NM_000251.2	Lynch syndrome
*MSH3*	5	NM_002439.4	Familiar Adenomatus Polyposis (FAP), endometrial cancer
*MUTYH*	1	NM_001128425.1	MUTYH-associated polyposis (MAP)
*NF2*	22	NM_000268.3	Neurofibromatosis type II
*NTRK1*	1	NM_002529.3	Congenital insensitivity to pain with anhidrosis (CIPA)
*PMS1*	2	NM_000534.4	Lynch syndrome
*PMS2*	7	NM_000535.5	Lynch syndrome
*POLD1*	19	NM_001308632.1	Polymerase Proofreading-Associated Polyposis (PPAP) syndrome
*POLE*	12	NM_006231.3	Colorectal cancer and facial dysmorphism, immunodeficiency, livedo, and short stature
*POLH*	6	NM_006502.2	Xeroderma pigmentosum
*PRF1*	10	NM_001083116.1	Familial hemophagocytic lymphohistiocytosis-2 (FHL2), aplastic anaemia
*PRSS1*	7	NM_002769.4	Hereditary pancreatitis, pancreatic cancer
*RAD54L*	1	NM_001142548.1	Lymphoma, Non-Hodgkin, Familial and Breast Ductal Carcinoma.
*RET*	10	NM_020975.4	Multiple Endocrine Neoplasia Type 2
*SAMD9*	7	NM_001193307.1	MIRAGE syndrome
*SBDS*	7	NM_016038.2	Shwachman-Diamond syndrome
*SDHD*	11	NM_003002.3	Carney-Stratakis syndrome
*SOS2*	14	NM_006939.3	Noonan Syndrome-9

*Associated syndrome according to ClinVar database (https://www.ncbi.nlm.nih.gov/clinvar/).

### Pathogenic/likely pathogenic variants

We found five pathogenic/likely pathogenic variants according to the American College of Medical Genetics and Genomics (ACMG) 2015 guidelines [36] in five unrelated patients. They included one missense variant and one nonsense variant in *BRCA1* gene, identified in two patients, one missense monoallelic variant in *MUTYH* gene, one nonsense variant in *MLH3* gene and one nonsense variant in *PMS1* gene, in three other patients (Table 3).

**Table 3:** Pathogenic/likely pathogenic variants.

Patient #	Gene	Chromosome	RefSeq	Exon	Nucleotide change	Amino acid change	dbSNP	Type of variant	Zygosity	Cancer Genome Interpreter	*Associated syndromes	Reported/novel	ACMG	Allele frequency (gnomAD)
P-7	*BRCA1*	17	NM_007294.2	5	c.287A>G	p.Asp96Gly	rs864622444	Missense	Heterozygous	Driver	Hereditary Breast and Ovarian Cancer Syndrome (HBOC)	LP (ClinVar)	LP	0.000001
P-44	*BRCA1*	17	NM_007294.2	10	c.1066C>T	p.Gln356Ter	rs80357215	Nonsense	Heterozygous	Passenger		P (ClinVar)	P	0.000001
P-24	*MUTYH*	1	NM_001128425.1	13	c.1187G>A	p.Gly396Asp	rs36053993	Missense	Heterozygous	Passenger	MUTYH-associated polyposis (MAP)	P (ClinVar)	P	0.0038
P-13	*MLH3*	5	NM_002439.4	3	c.3343C>T	p.Arg1115Ter	rs749297558	Nonsense	Heterozygous	Driver	Familiar Adenomatus Polyposis (FAP), endometrial cancer	Novel	**P	0.0001
P-52	*PMS1*	2	NM_000534.4	9	c.1826G>A	p.Trp609Ter	NR	Nonsense	Heterozygous	Driver	Lynch syndrome	Novel	**P	-

Abbreviations: P, pathogenic; LP, likely pathogenic; ClinVar (https://www.ncbi.nlm.nih.gov/clinvar/);

*Associated syndrome according to ClinVar database;

**Fulfilled 2015 ACMG criteria for pathogenic/likely pathogenic variants [36]: PVS1: (null variant nonsense, frameshift mutation, canonical +- 2 splice sites, initiation codon, single or multiexon deletion) + PM2 (absent from controls or at extremely low frequency if recessive) + PP3 (multiple lines of computational evidence support a deleterious effect on the gene or gene product).

**Fig 3:**
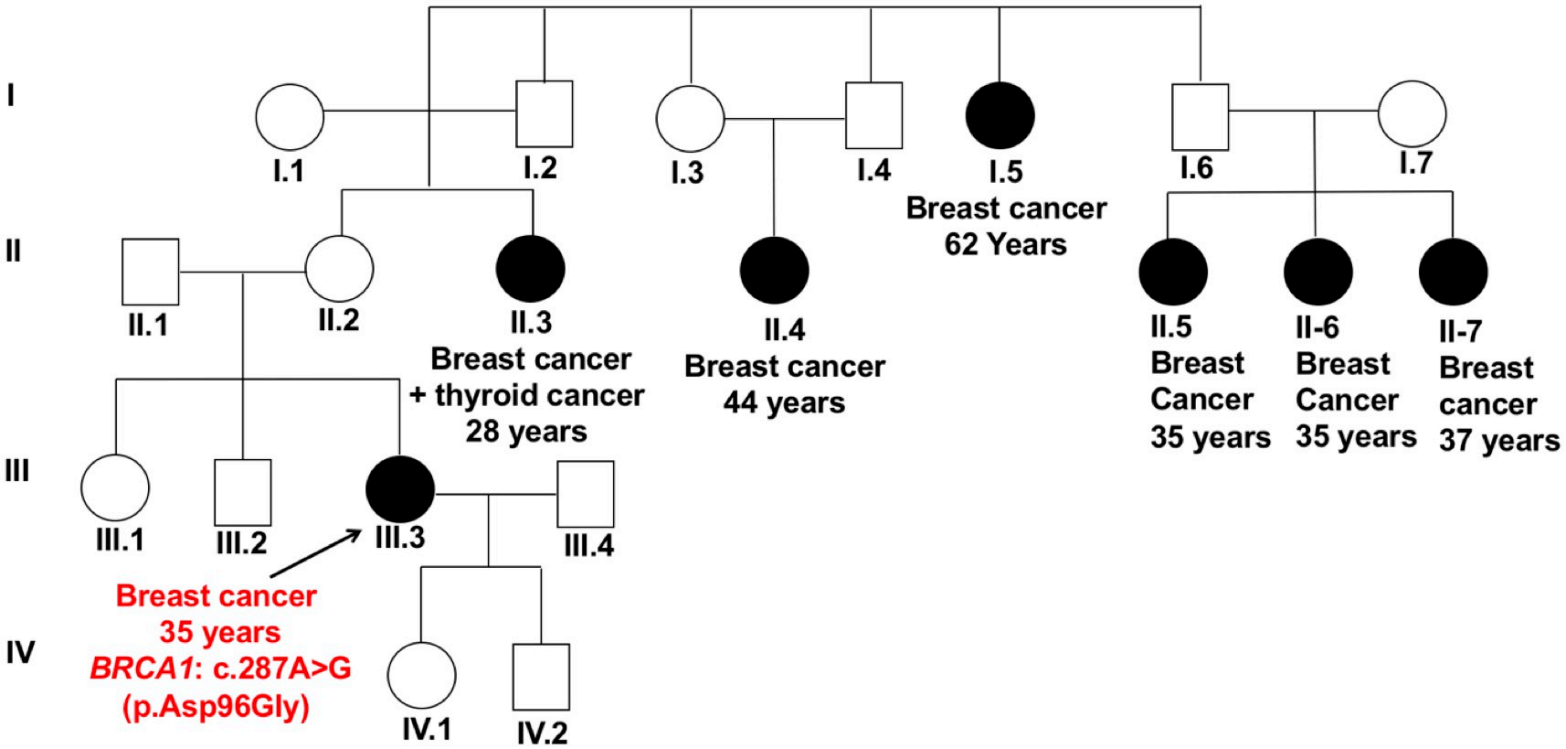
Pedigree of patient P-7 carrying the missense variant c.287A>G (p.Asp96Gly) in *BRCA1* gene. The patient was diagnosed with unilateral breast cancer at age 35 years, with her maternal aunt affected by breast cancer and thyroid cancer at age 28 years old, as well as other family members affected with breast cancer. The symbols used are proband (arrow), male (box), female (circle), affected female (black fill).

The *BRCA1* missense variant c.287A>G (p.Asp96Gly) lies in exon 5 at a mutational hotspot and a critical well-established functional domain, and results in loss of normal protein function and cellular response to DNA damage. The patient harboring this variant (P-7) was diagnosed with unilateral, TNBC at age 35 years. The relevant family history included her maternal aunt affected by breast cancer and thyroid cancer at a young age (28 years), and five other family members affected by breast cancer (Fig 3).

**Fig 4:**
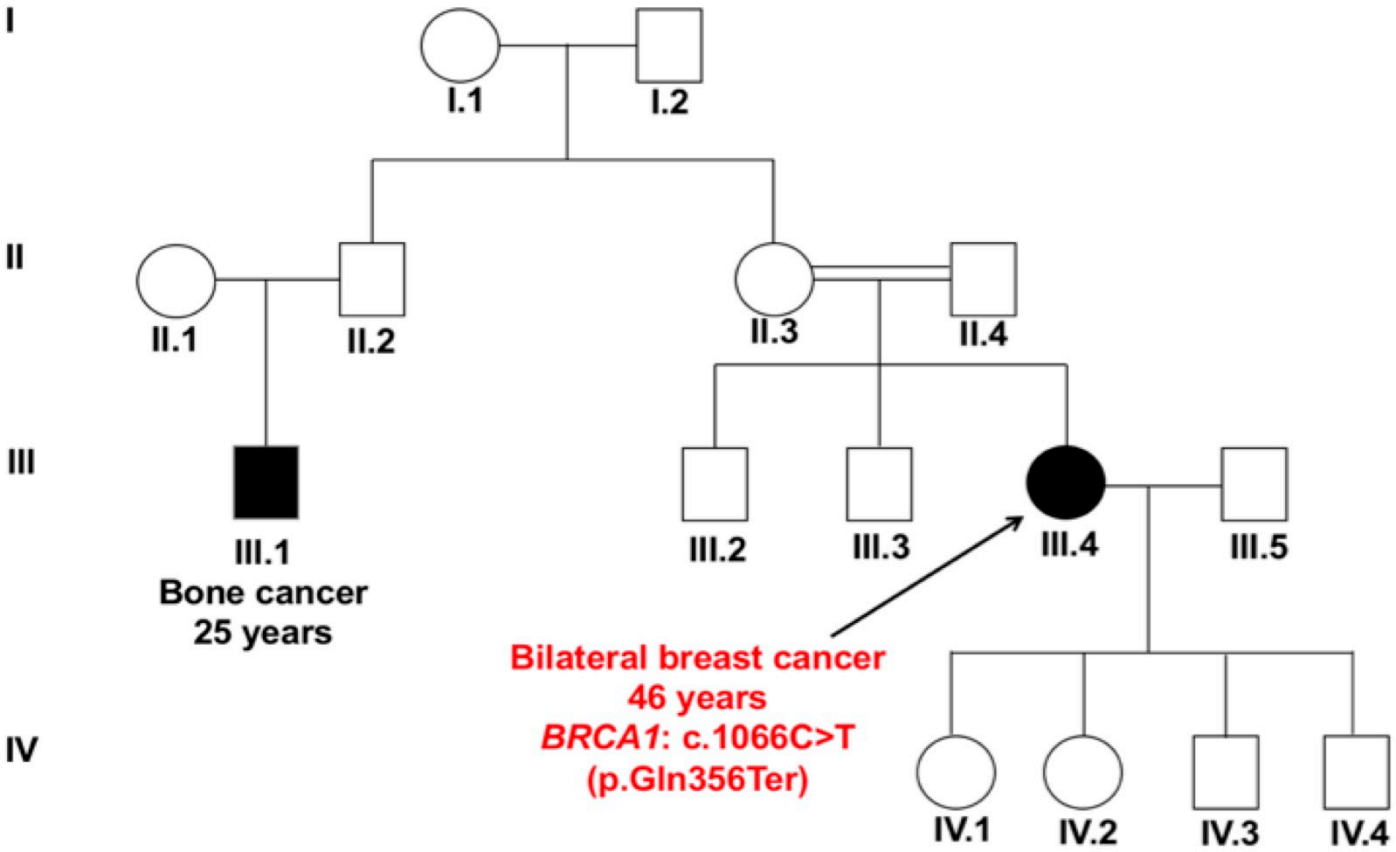
Pedigree of patient P-44 carrying the nonsense variant c.1066C>T (p.Gln356Ter) in *BRCA1* gene. The patient who descended from consanguineous parents was diagnosed with bilateral breast cancer at age 46 years and her maternal cousin was affected by bone cancer. The symbols used are proband (arrow), male (box), female (circle), affected male and female (black fill), consanguinity (double lining).

The *BRCA1* nonsense variant c.1066C>T (p.Gln356Ter) lies in exon 10 and results in truncated non-functional protein in the domain of the BRCA repeats, interfering with the cellular response to DNA damage. This variant was detected in a patient (P-44) who descended from consanguineous parents, and was diagnosed with bilateral breast cancer at age 46 years without a family history of breast cancer, but her maternal cousin was affected by bone cancer (Fig 4).

**Fig 5:**
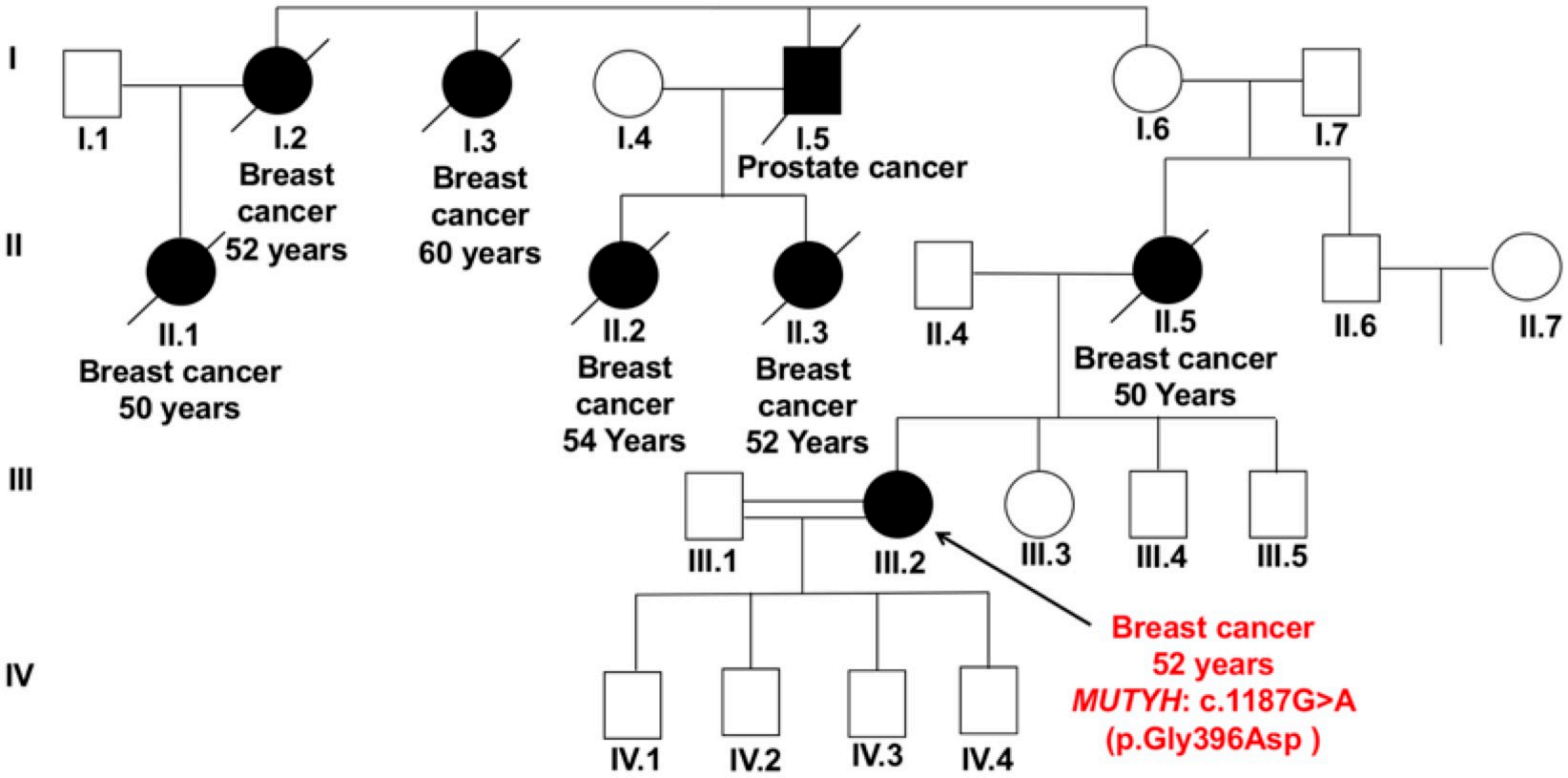
Pedigree of patient P-24 carrying the missense variant c.1187G>A (p.Gly396Asp) in *MUTYH* gene. The patient was diagnosed with unilateral breast cancer at age 52 years with a strong family history of her mother died due to breast cancer at age 50 years, two other family members died due to breast cancer, and a family member died due to prostate cancer (all from the maternal branches) as well as three other family members died due to breast cancer. The symbols used are proband (arrow), male (box), female (circle), affected male and female (black fill), deceased (line through), consanguinity (double lining).

The *MUTYH* missense variant c.1187G>A (p.Gly396Asp) lies in exon 13 at a mutational hotspot and a critical functional domain, and results in loss of normal protein function. The patient harboring this variant (P-24) was diagnosed with unilateral breast cancer at age 52 years. The relevant family history included her mother and other family members died due to breast cancer and a family member died due to prostate cancer (Fig 5).

**Fig 6:**
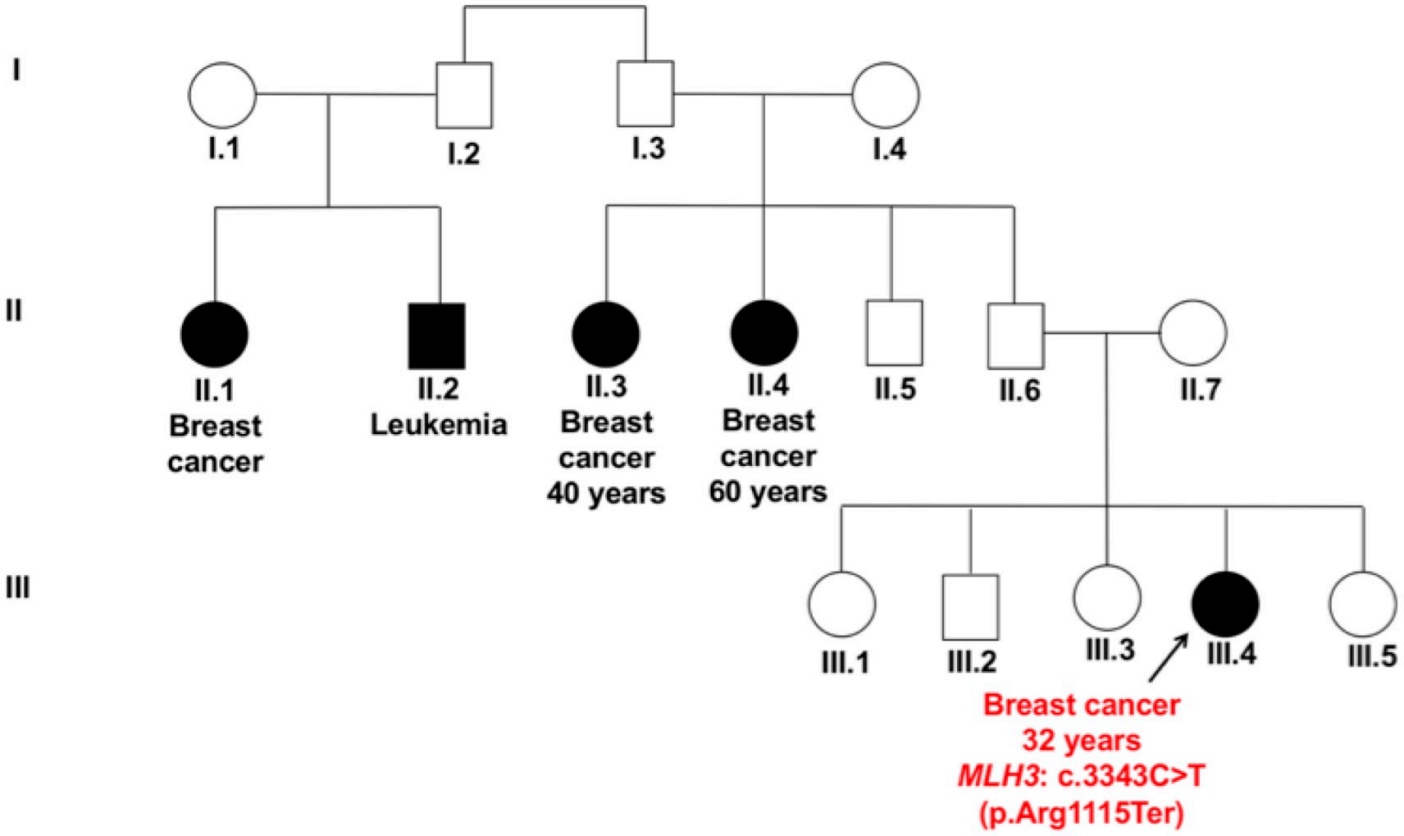
Pedigree of patient P-13 carrying the nonsense variant c.3343C>T (p.Arg1115Ter) in *MLH3* gene. The patient was diagnosed with unilateral breast cancer at age 32 years with her two paternal aunts affected by breast cancer and other family members affected by breast cancer and/or leukaemia. The symbols used are proband (arrow), male (box), female (circle), affected male and female (black fill).

The *MLH3* nonsense variant c.3343C>T (p.Arg1115Ter) lies in exon 3 and results in truncated non-functional protein. The patient harboring this variant (P-13) was diagnosed with unilateral breast cancer, and showed two paternal aunts affected by breast cancer, and other family members affected by breast cancer and/or leukemia (Fig 6).

**Fig 7:**
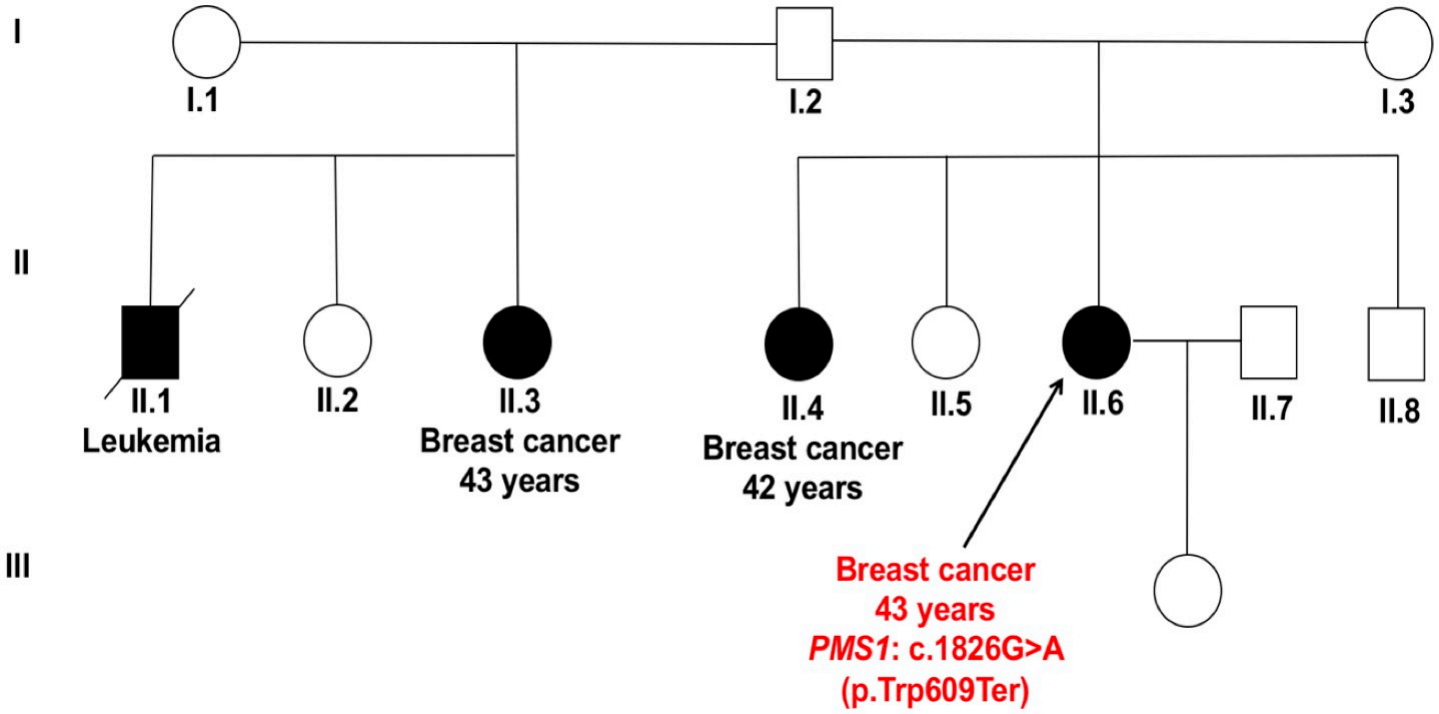
Pedigree of patient P-52 carrying the nonsense variant c.1826G>A (p.Trp609Ter) in *PMS1* gene. The patient was diagnosed with unilateral breast cancer at age 43 years with a strong family history of her two sisters affected by breast cancer, as well as a family member who died due to leukaemia. The symbols used are proband (arrow), male (box), female (circle), affected male and female (black fill).

The *PMS1* nonsense variant c.1826G>A (p.Trp609Ter) lies in exon 9 and results in truncated non-functional protein. Patient P-52 who carried this variant was diagnosed with unilateral breast cancer, and showed two sisters affected by breast cancer and a family member died due to leukemia (Fig 7).

### Variants of uncertain significance

We also found several variants of uncertain significance (VUS) in different cancer predisposition-genes among twenty five patients (46.3%) (Table 4). Of them, two missense variants namely (p.Ile1307Lys) in *APC* gene and c.1441G>T (p.Asp481Tyr) in *CHEK2* gene were classified as likely benign according to the ACMG criteria, and as variants with conflicting interpretation of pathogenicity in the ClinVar database. Bioinformatics analysis using CADD and REVE tools predicted both variants to be benign. Two other interesting variants were identified. The missense variant c.5671C>A in *FAT1* gene, was reported as likely pathogenic in ClinVar database and as VUS according to ACMG/AMP criteria. However, this variant was predicted to be benign by CADD and REVEL tools. The missense variant c.2074A>G in *POLH* gene was reported as pathogenic in ClinVar database and as VUS according to ACMG/AMP criteria. This variants was predicted to be deleterious by REVEL (score 0.62). Furthermore, the variant c.661G>A (p.Ala221Thr) in *PRSS1* gene was not previously reported in any database and may be considered as novel. This variant was predicted to be deleterious by REVEL (score 0.86). Several other variants with uncertain significance were predicted to be deleterious by REVEL (bold), (Table 4).

**Table 4:** Variants of uncertain significance.

Patient #	Gene	Chromosome	RefSeq	Exon	Nucleotide change	Amino acid change	dbSNP	Type of variant	Zygosity	ClinVar	ACMG	Allele frequency (gnomAD)	CADD Score	CADD PHRED	REVEL
P-3	*APC*	5	NM_000038.5	18	c.6674C>G	p.Ser2225Cys	rs759307079	Missense	Heterozygous	VUS	VUS	0.000008	3.5306	24.8	0.46
	*POLE*	12	NM_006231.3	12	c.1184G>A	p.Gly395Glu	rs546499094	Missense	Heterozygous	VUS	VUS	0.00022	3.7166	25.5	0.37
	***SDHD***	**11**	**NM_003002.3**	**4**	**c.335C>T**	**p.Thr112Ile**	**rs199869408**	**Missense**	**Heterozygous**	**VUS**	**VUS**	**0.000007**	**3.3275**	**24.2**	**0.87**
P-5	*POLD1*	19	NM_001308632.1	19	c.2638G>A	p.Asp880Asn	rs373650022	Missense	Heterozygous	VUS	VUS	0.00057	2.6045	22.6	0.13
P-6	***APC***	**5**	**NM_000038.5**	**16**	**c.3484T>C**	**p.Tyr1162His**	**rs1210397302**	**Missense**	**Heterozygous**	**VUS**	**NR**	**0.000004**	**3.9313**	**26.5**	**0.67**
	***NTRK1***	**1**	**NM_002529.3**	**17**	**c.2326G>T**	**p.Asp776Tyr**	**NR**	**Missense**	**Heterozygous**	**NR**	**VUS**	**NR**	**4.3851**	**31**	**0.78**
	*PMS1*	2	NM_000534.4	2	c.17C>T	p.Ala6Val	rs747835603	Missense	Heterozygous	VUS	VUS	0.00006	3.2111	23.9	0.45
P-7	***ATM***	**11**	**NM_000051.3**	**58**	**c.8521G>T**	**p.Asp2841Tyr**	**rs786203013**	**Missense**	**Heterozygous**	**VUS**	**VUS**	**0.000004**	**4.4349**	**31**	**0.84**
P-8	*APC*	5	NM_000038.5	16	c.3387G>T	p.Leu1129Phe	rs730881225	Missense	Heterozygous	VUS	VUS	0.000007	2.8531	23.1	0.38
	*MSH3*	5	NM_002439.4	21	c.2992A>G	p.Ile998Val	rs548030451	Missense	Heterozygous	VUS	VUS	0.000001	3.1325	23.7	0.42
	***RET***	**10**	**NM_020975.4**	**14**	**c.2522C>T**	**p.Pro841Leu**	**rs149891333**	**Missense**	**Heterozygous**	**VUS**	**VUS**	**0.00015**	**3.3856**	**24.4**	**0.81**
P-9	***SBDS***	**7**	**NM_016038.2**	**5**	**c.664G>C**	**p.Glu222Gln**	**rs371133001**	**Missense**	**Heterozygous**	**VUS**	**VUS**	**0.000057**	**3.6287**	**25.1**	**0.79**
P-11	*PMS2*	7	NM_000535.5	13	c.2186_2187delTC	p.Leu729fs	rs587779335	Frameshift deletion	Heterozygous	VUS	NR	0.0084	5.3280	33	0.46
P-12	*FAM175A*	4	NM_139076.2	8	c.761T>C	p.Ile254Thr	NR	Missense	Heterozygous	VUS	VUS	0.000046	2.7557	22.9	0.11
	*FAT1*	4	NM_005245.3	5	c.3904A>G	p.Ile1302Val	rs752677928	Missense	Heterozygous	NR	LB	0.000001	3.2312	23.9	0.34
P-18	***PRF1***	**10**	**NM_001083116.1**	**3**	**c.1456G>T**	**p.Asn34Ser**	**NR**	**Missense**	**Heterozygous**	**NR**	**VUS**	**NR**	**3.3134**	**24.2**	**0.73**
P-20	*ERCC4*	16	NM_005236.2	5	c.799C>T	p.Arg267Cys	rs373570729	Missense	Heterozygous	VUS	VUS	0.000046	3.3005	24.1	0.47
	*FAT1*	4	NM_005245.3	5	c.13013A>C	p.Asp4338Ala	NR	Missense	Heterozygous	NR	VUS	NR	3.7347	25.5	0.22
P-22	***ATM***	**11**	**NM_000051.3**	**21**	**c.3083T>G**	**p.Leu1028Arg**	**rs1555085776**	**Missense**	**Heterozygous**	**VUS**	**VUS**	**NR**	**4.0266**	**27.2**	**0.72**
	*CBL*	11	NM_005188.3	13	c.2107C>T	p.Pro703Ser	rs1229733932	Missense	Heterozygous	VUS	VUS	0.000004	3.7162	25.5	0.39
P-24	*APC*	5	NM_000038.5	16	c.3920T>A	p.Ile1307Lys	rs1801155	Missense	Heterozygous	Conflicting	LB*	0.00116	0.7073	8.522	0.23
	***MSH2***	**2**	**NM_000251.2**	**3**	**c.458C>G**	**p.Ser153Cys**	**rs766349734**	**Missense**	**Heterozygous**	**VUS**	**VUS**	**0.000001**	**2.5038**	**22.4**	**0.57**
P-26	***FANCI***	**15**	**NM_001113378.1**	**8**	**c.3419C>T**	**p.Thr1140Ile**	**rs142969866**	**Missense**	**Heterozygous**	**VUS**	**VUS**	**0.000028**	**3.1649**	**23.8**	**0.57**
	***PMS1***	**2**	**NM_000534.4**	**8**	**c.889C>G**	**p.Leu297Val**	**NR**	**Missense**	**Heterozygous**	**NR**	**VUS**	**NR**	**2.1852**	**20.8**	**0.57**
P-29	*CHEK2*	22	NM_007194.3	13	c.1441G>T	p.Asp481Tyr	rs200050883	Missense	Homozygous	Conflicting	LB*	0.00004	3.8240	25.9	0.34
P-30	*FANCI*	15	NM_001113378.1	22	c.2266T>C	p.Tyr756His	rs752028405	Missense	Heterozygous	VUS	VUS	0.000016	3.8518	26.1	0.27
	*FANCM*	14	NM_020937.3	21	c.5569G>A	p.Val1857Met	rs144008013	Missense	Heterozygous	VUS	VUS	0.00043	3.5828	25	0.37
P-32	*FAT1*	4	NM_005245.3	10	c.5671C>A	p.Pro1891Thr	rs185078412	Missense	Heterozygous	LP	VUS*	0.00004	3.1506	23.7	0.46
P-34	*ERCC3*	2	NM_000122.1	5	c.569A>G	p.Gln190Arg	NR	Missense	Heterozygous	NR	VUS	NR	2.4481	22.3	0.41
P-36	*RAD54L*	1	NM_001142548.1	6	c.319C>T	p.Arg107Trp	rs141019694	Missense	Heterozygous	VUS	NR	0.000057	3.7768	25.7	0.26
P-42	***CTRC***	**1**	**NM_007272.2**	**6**	**c.518T>A**	**p.Leu173Gln**	**NR**	**Missense**	**Homozygous**	**NR**	**VUS**	**NR**	**4.0006**	**27**	**0.97**
	***PRSS1***	**7**	**NM_002769.4**	**5**	**c.661G>A**	**p.Ala221Thr**	**NR**	**Missense**	**Homozygous**	**NR**	**NR**	**NR**	**3.7936**	**25.8**	**0.86**
P-46	*MSH3*	5	NM_002439.4	12	c.1655C>T	p.Thr552Ile	rs749862056	Missense	Heterozygous	VUS	VUS	0.000036	4.1652	28.3	0.5
	***NF2***	**22**	**NM_000268.3**	**12**	**c.1264G>A**	**p.Glu422Lys**	**rs2066864084**	**Missense**	**Heterozygous**	**VUS**	**VUS**	**NR**	**3.9873**	**26.9**	**0.69**
	*PMS1*	2	NM_000534.4	12	c.2611C>T	p.Arg871Cys	rs143554211	Missense	Heterozygous	VUS	VUS	0.000016	4.1425	28.1	0.47
	***SOS2***	**14**	**NM_006939.3**	**10**	**c.1537G>A**	**p.Glu513Lys**	**NR**	**Missense**	**Heterozygous**	**NR**	**VUS**	**NR**	**3.9195**	**26.5**	**0.65**
P-47	*KIF1B*	1	NM_015074.3	13	c.1274A>G	p.Glu425Gly	NR	Missense	Heterozygous	NR	VUS	NR	2.6053	22.6	0.18
P-49	*FLCN*	17	NM_144997.5	7	c.740A>C	p.Asp247Ala	NR	Missense	Heterozygous	NR	VUS	NR	3.5770	25	0.72
P-50	*ERCC3*	2	NM_000122.1	2	c.231G>T	p.Trp77Cys	rs201635630	Missense	Heterozygous	NR	NR	0.000004	4.3452	29.9	0.72
P-51	***POLH***	**6**	**NM_006502.2**	**12**	**c.2074A>G**	**p.Thr692Ala**	**rs199562456**	**Missense**	**Heterozygous**	**P**	**VUS** *	**0.000057**	**1.0700**	**12.46**	**0.62**
	***SAMD9***	**7**	**NM_001193307.1**	**2**	**c.2395G>C**	**p.Asp799His**	**NR**	**Missense**	**Heterozygous**	**NR**	**VUS**	**NR**	**3.5583**	**24.9**	**0.88**
P-52	***SOS2***	**14**	**NM_006939.3**	**20**	**c.3275C>T**	**p.Pro1092Leu**	**rs1442962879**	**Missense**	**Heterozygous**	**VUS**	**NR**	**0.000007**	**4.1241**	**28**	**0.57**

Abbreviations: VUS, variant of uncertain significance; conflicting, conflicting interpretation of pathogenicity; P, pathogenic; LP, likely pathogenic, LB, likely benign; NR, not reported; ClinVar (https://www.ncbi.nlm.nih.gov/clinvar/); ACMG, American College of Medical Genetics.

*Variants did not fulfil 2015 ACMG guidelines as pathogenic or likely pathogenic [36] but were classified as VUS or pathogenic/likely pathogenic in the ClinVar database. CADD, Combined Annotation Dependent Depletion; REVEL combines scores from 13 different individual tools for the prediction of pathogenicity; variants predicted to be deleterious by REVEL (bold).

In addition, fourteen patients (25.9%) were found to carry variants in two or more cancer susceptibility genes. Particularly, four patients carried pathogenic/likely pathogenic or potential pathogenic variants together with distinct other VUS. Patient P-7 harboring the likely pathogenic variant c.287A>G (p.Asp96Gly) in *BRCA1* gene was found to carry a VUS in *ATM* gene. Patient P-24 harboring the pathogenic variant c.1187G>A (p.Gly396Asp) in *MUTYH* gene was found to carry two VUS in *APC* and *MSH2* genes. Patient P-52 harboring the potentially pathogenic variant in *POLH* gene was found to carry a VUS in *SAMD9* gene. Patient P-52 harboring the pathogenic variant c.1826G>A (p.Trp609Ter) in *PMS1* gene was found to carry a VUS in *SOC2* gene.

## Discussion

Traditionally, genetic testing for breast cancer has been restricted to high-risk predisposition genes, such as *BRCA1* and *BRCA2*. (*BRCA1/2*). Although germline variants in *BRCA1/2* confer a higher risk of hereditary breast cancer [14–18], many breast cancer patients test negative for variants in these two genes [24–28] and additional non-*BRCA* genes, particularly those participating in the DNA repair mechanisms, have been identified as predisposing genes for breast cancer [27]. Deficiency in DNA repair is the underlying cause of genomic instability that contribute to tumorigenesis and has implications for therapy in a variety of cancers [38–40]. In the current era of NGS, the usage of multigene panels provides more information on a large number of cancer susceptibility genes, allowing for more accurate risk stratification and tailored cancer care. A number of recent studies have shown a higher rate of variants in non-*BRCA1/2* genes, and also found differences in the spectrum and prevalence of germline variants in cancer susceptibility gene in breast cancer patients among ethnicities [31–33]. Since multigene panel-testing data are still missing in the Bahraini population, the current study investigated germline variants in a cohort of 54 Bahraini women with a positive personal and/or family history of breast cancer using an NGS-based gene-panel covering 180 cancer susceptibility genes.

Sequencing data revealed 47 germline variants in 29 patients (Fig 1 and Table 2). Of all patients, five were pathogenic or likely pathogenic variant carriers (Fig 2 and Table 3) including two patients with variants in BRCA1 gene and three patients with variants in non-*BRCA1/2* genes namely *MUTYH*, *MLH3* and *PMS1* genes.

*BRCA1* gene variants included a loss-of-function missense variant (c.287A>G; p.Asp96Gly) (Fig 3) [41], and a truncating nonsense variant (c.1066C>T; p.Gln356Ter) (Fig 4), detected in two unrelated patients. *BRCA1* is a key DNA repair gene that plays an important role in maintaining genome stability by repairing double-strand DNA breaks through a homologous recombination repair (HRR) pathway [42]. *BRCA1* also acts as a tumor suppressor gene, which is frequently mutated in familial breast and ovarian cancers. Approximately 11.2% of patients with TNBC carry germline variants in *BRCA1* or *BRCA2* genes, and they are usually diagnosed at a younger age and have a positive family history of breast cancer [43]. TNBC is characterized by the lack of expression of estrogen receptor (ER), progesterone receptors (PR) and human epidermal growth factor receptor 2 (HER2/neu), and is associated with poor prognosis [43]. Detection of variants in *BRCA1/2* genes is clinically important in breast cancer patients. Cancer cells with harmful variants in *BRCA1/2* are unable to repair DNA double-strand breaks and they are heavily rely on single-strand break repair pathways [44]. The PARP enzyme is involved in repairing single-strand DNA breaks, and PARP inhibitors result in the accumulation of unrepaired single-strand breaks, leading to cell death. Therefore, patients with *BRCA1/2* variants were predicted to benefit from PARP inhibitors such as Talazoparib and Olaparib [20–22]. In recent clinical trials, PARP inhibitors were also approved for TNBC patients, which showed progression-free survival benefit when compared to chemotherapy [45]. In addition, PARP inhibitors were found to be effective options for treatment of patients with advanced or metastatic TNBC [45, 46].

The prevalence of *BRCA1/2* variants differs according to ethnic, geographical and other factors. In a systematic review by Armstrong et al,. [47], the prevalence of *BRCA1/2* variants was found to vary widely within key clinical and demographic subgroups across countries (Australia, Canada, France, Germany, Israel/Palestine, Italy, Japan, Russia, South Korea, Spain, United Kingdom, and United States). In other ethnic groups, a relatively high rate of *BRCA1/2* germline variants was reported in familial breast cancer in Turkey (9.1%) [48], and in Chinese patients with hereditary breast/ovarian cancer (9.4%) [49]. Whereas, a greater rate of *BRCA1/2* germline variants was reported in familial breast cancer patients from Lebanon (15.5%) [50], and Egypt (19.8% for *BRCA1* and 30.6% for *BRCA2*) [33]. Data from the Gulf Cooperation Council (GCC) states have shown higher rates of *BRCA1/2* variants in breast cancer patients. For instance, the prevalence of *BRCA1* and *BRCA2* variants was 89.3% and 14.3% respectively in Saudi Arabia [51], and the overall prevalence of *BRCA1/2* variants was 39.6% in Qatar [52]. A previous study in Bahrain by Al Hannan et al., [34], which investigated the frequency of *BRCA1/2* germline variants in familial breast cancer women, showed that only one patient (1/25, 4%) was a carrier of *BRCA1* gene variant and one patient was a *BRCA2* variant carrier. In our study, the prevalence of *BRCA1* variants was 3.7%, which is almost similar to Al Hannan et al., study [34].

Three other germline variants were found in non-*BRCA* genes: *MUTYH*, *MLH3* and *PMS1* genes. In *MUTYH* gene, a deleterious missense variant (c.1187G>A; p.Gly396Asp) was detected in a breast cancer patients with a strong family history of breast cancer (Fig 5). *MUTYH* (human MutY homolog) is a DNA repair gene, which encodes DNA glycosylase involved in base excision repair during DNA replication and DNA damage repair [53]. Due to the essential role of *MUTYH* gene in DNA repair, it has been implicated in many other cancers [54]. Specifically, biallelic pathogenic germline variants in *MUTYH* gene have been associated with *MUTYH*-associated polyposis, an autosomal recessive condition which increases the risk of colorectal cancer [55]. It is generally believed that recessive genes are not pathogenic in heterozygotes. The association between monoallelic variants in *MUTYH* gene and risk of breast cancer is controversial, with some studies showing no link [56, 57] and others reporting an association [58, 59]. In recent studies, *MUTYH*-monoallelic germline variants have been reported in patients with early-onset or familial breast cancer [60] and in *BRCA1/2* negative breast cancer patients [61–63]. In particular, the identified *MUTYH* c.1187G>A (p.Gly396Asp) variant in our study was one of the prevalent variants in Dutch patients with adenomatous polyposis, accounting for about 75% of all *MUTYH* pathogenic variants [64]. Interestingly, this specific variant was previously reported in Egyptian patients with familial breast cancer [33], and in Dutch families with breast cancer and colorectal cancer [59]. Monoallelic variants in *MUTYH* gene may act as low-penetrance breast cancer risk and could contribute to breast cancer development in synergy with additional risk factors such as age, ethnicity, or even environmental and lifestyle factors. The current guidelines of the NCCN for Genetic/Familial High- Risk Assessment: Breast, Ovarian, and Pancreatic (version 2.2022, accessed on March 2022) recommended including *MUTYH-*monoallelic variants as one of the low-penetrance genes for multigene testing. Nevertheless, further studies are required to confirm the association between *MUTYH-*monoallelic variants and risk of breast cancer, which can provide additional knowledge for assessing the patient’s risk and the potential of developing a new therapeutic target [39].

The other two germline variants in non *BRCA1/2* genes in our study were truncating variants, found in two mismatch repair (MMR)-related genes namely c.3343C>T (p.Arg1115Ter) in *MLH3* gene and c.1826G>A (p.Trp609Ter) in *PMS1* gene (Figs 6 and 7). The MMR system is involved in the maintenance of genomic integrity during DNA replication and after meiotic recombination. Mutated MMR genes cause microsatellite instability (MSI) and replication of error-positive phenotype [65, 66]. Studies have shown that germline variants in MMR genes increase the risk of colorectal cancer and other cancers [67].

*MLH3* gene encodes a protein that functions as a heterodimer with other MMR genes, and *PMS1* gene encodes a protein that forms a heterodimer with *MLH1* and *PMS2*, products of MMR genes involved in Lynch syndrome (nonpolyposis colorectal cancer, HNPCC) [68]. Both *MLH3* and *PMS1* genes are suspected to play a role in Lynch syndrome, but the clinical significance of variants in these genes is less clear [69]. Recently, germline variants in *MLH3* gene have been reported in Finnish families with adenomatous polyposis who also exhibited breast cancer [70], and in Chinese patients with breast/ovarian cancer [32]. In addition, unique germline variants in *PMS1* gene have been found in patients with breast cancer and in patients with breast/ovarian syndrome [71, 72].

Although germline variants in MMR genes are rare in breast cancer and the values of these genes are unknown with respect to breast cancer risk, it has been shown that breast cancer carriers of variants in these genes might have worse survival and some of them might benefit from immunotherapy [73]. Thus, the advantage of MMR genes in breast cancer genetic testing cannot be completely ruled out.

It has been suggested that some pathogenic variants linked with cancer which are common in the European population show lower association with cancer in other ethnic groups such as people of Arab descent [74]. In a previous study in Saudi Arabian breast cancer patients, 37 potential variants in 25 breast cancer risk associated genes other than *BRCA1/2* were identified including variants in *MLH1*, *MLH3* genes [75]. Other studies in Saudi Arabian breast cancer patients showed that rare pathogenic variants in *MUTYH* gene and other MMR genes such as *MLH1*, *MSH2*, *MSH6* may potentially increase the risk of breast cancer [76].https://www.ncbi.nlm.nih.gov/pmc/articles/PMC10259259/ In addition, pathogenic variants in *MUTYH* gene and MMR genes were also reported in breast cancer patients from the UAE [77].

Clinical intervention and management for breast cancer patients with *BRCA1/2* variants are well established and widely applied in clinical practice. However, low-risk genes such as *MLH3* and *PMS1* are not available in the management guidelines, where variants in low-penetrance genes are very rare and correlate with < 2-fold risk of developing breast cancer [78]. In this sense, medical decisions should be based on combined personal and family history of breast cancer patients in pre- and post-genetic counseling.

We also found a number of variants of uncertain significance (VUS) in different cancer predisposing-genes (Table 4). Of them, two missense variants namely c.3920T>A (p.Ile1307Lys) in *APC* gene and c.1441G>T (p.Asp481Tyr) in *CHEK2* gene were classified as likely benign according to the ACMG criteria and as variants with conflicting interpretation of pathogenicity in the ClinVar database. Notably, in our study both variants were predicted to be benign by CADD and REVEL tools. Moreover, the missense variants c.5671C>A (p.Pro1891Thr) in *FAT1* gene and c.2074A>G (p.Thr692Ala) in *POLH* gene were reported as VUS according to the ACMG criteria, but as likely pathogenic and pathogenic respectively in the ClinVar database. In our bioinformatic analysis, the *POLH* c.2074A>G variant was predicted to be deleterious by REVEL (score 0.62). Particularly this specific variant was previously detected in a Lebanese breast cancer family [50]. Therefore, it deserves further investigation in a larger cohort with additional functional studies to assess its impact on breast cancer. We also found a novel missense variant (c.661G>A; p.Ala221Thr) in *PRSS1* gene, which was not previously reported in any database. It was predicted to be deleterious by REVEL (score 0.86). However, there are no established functional studies to support the impact of these variants on protein function and further studies are needed to assess their consequences.

Interpretation of VUS can cause a great deal of uncertainty with inconclusive and frustrating results for patients, especially in case of family history of cancers. Currently there is a lack of consensus management guidelines for clinicians on VUS [29]. However, reporting these variants may contribute to the knowledge of unclassified variants in breast cancer and gives us the opportunity to conduct further research to clarify their clinical significance.

Fourteen breast cancer patients (25.9%) in our study were found to carry variants in two or more cancer-predisposing genes. In particular, three patients were found to harbor pathogenic or likely pathogenic variants with other VUS. It was not surprising to detect more than one variant through analysis with high-throughput sequencing technology, and the co-presence of these variants can be normal polymorphism in the population with no biological significance.

Strengths of this study include the innovative use of NGS analysis in an underrepresented population, alongside multiple bioinformatics tools that assisted us with the *in silico* characterization of detected variants. However, our study was limited by the small number of breast cancer patients, and further studies in larger sample sizes are warranted. Additionally, the individuals included in our study represent unrelated patients and there is no prior knowledge about the status of germline variant in their family members. Consequently, further studies are required to assess their possible risk and their potential benefit of preventative management. Mainly, exploration of the impact of *MLH3* and *PMS1* germline variants in breast cancer would require further segregation analysis and screening tests such as MSI test in larger breast cancer cohorts.

## Conclusions

Five pathogenic/likely pathogenic variants were reported here for the first time in Bahraini women with breast cancer. These variants were found in genes that play critical roles in DNA repair, including two variants in *BRCA1* gene and three variants in non-*BRCA1/2* genes namely *MUTYH*, *MLH3* and *PMS1*. Several other variants of uncertain significance were detected, and some of them were found together with the pathogenic/likely pathogenic variants. To the best of our knowledge, this is the first application of NGS using a comprehensive cancer-predisposition gene-panel in Bahrain women with breast cancer. Our findings show that multigene testing can yield additional genomic information on low-penetrance genes and provide valuable epidemiological information for future studies. Our findings also highlight the importance of genetic testing, and an NGS-based multigene analysis may be applied supplementary to traditional genetic counseling.

## References

[1] Arnold M, Morgan E, Rumgay H, Mafra A, Singh D, Laversanne M, et al. Current and future burden of breast cancer: Global statistics for 2020 and 2040. The Breast. 2022;66: 15–23. doi: 10.1016/j.breast.2022.08.010 36084384 PMC9465273

[2] Larsen MJ, Thomassen M, Gerdes AM, Kruse TA. Hereditary breast cancer: Clinical, pathological and molecular characteristics. Breast Cancer (Auckl). 2014;8: 145–155. doi: 10.4137/BCBCR.S18715 25368521 PMC4213954

[3] Rebbeck TR, Friebel TM, Friedman E, Hamann U, Huo D, Kwong A, et al., and EMBRACE, and GEMO Study Collaborators, and HEBON. Mutational spectrum in a worldwide study of 29,700 families with BRCA1 or BRCA2 mutations. Hum Mutat. 2018;39: 593–620. 29446198 10.1002/humu.23406 PMC5903938

[4] Central Intelligence Agency. September 27, 2021 –via CIA.gov.

[5] Rodriguez-Flores JL, Fakhro K, AgostoPerez F, Ramstetter MD, Arbiza L, Vincent TL, et al. Indigenous Arabs are descendants of the earliest split from ancient Eurasian populations. Genome Res. 2016;26: 151–162. doi: 10.1101/gr.191478.115 26728717 PMC4728368

[6] Bahri R, Halima AB, Ayadi I, Esteban E, Alfadhli SM, Rebai A, et al. Genetic position of Bahrain natives among wider Middle East populations according to Alu insertion polymorphisms. Ann Hum Biol. 2013;40(1): 35–40. doi: 10.3109/03014460.2012.728622 23039013

[7] Garcia-Bertrand R, Simms TM, Cadenas AM, Herrera RJ. United Arab Emirates: phylogenetic relationships and ancestral populations. Gene. 2014;533: 411–419. doi: 10.1016/j.gene.2013.09.092 24120897

[8] Tadmouri GO, Nair P, Obeid T, Al Ali MT, Al Khaja N, Hamamy HA. Consanguinity and reproductive health among Arabs. Reprod Health. 2009;6: 17. doi: 10.1186/1742-4755-6-17 19811666 PMC2765422

[9] Hamadeh RR, Abulfatih NM, Fekri MA, Al-Mehza HE. Epidemiology of breast cancer among Bahraini women. Data from the Bahrain Cancer Registry. Sultan Qaboos Univ Med J. 2014;14(2): e176–e182. 24790739 PMC3997533

[10] Ravichandran K, Al-Zahrani AS. Association of reproductive factors with the incidence of breast cancer in Gulf Cooperation Council countries. EMHJ. 2009;15(3): 612–21. 19731777

[11] National Genome Center https://www.moh.gov.bh/GenomeProject?lang=en#:~:text=The%20Bahrain%20Genome%20Project%20aims,so%20called%20%E2%80%9Cpersonalized%20medicine%E2%80%9D.

[12] Cancer, Collaborative Group on Hormonal Factors in Breast Cancer (CGoHFiB) Familial breast cancer: collaborative reanalysis of individual data from 52 epidemiological studies including 58 209 women with breast cancer and 101 986 women without the disease. The Lancet. 2001;358: 1389–1399. doi: 10.1016/S0140-6736(01)06524-2 11705483

[13] Brewer HR, Jones ME, Schoemaker MJ, Ashworth A, Swerdlow AJ. Family history and risk of breast cancer: an analysis accounting for family structure. Breast Cancer Res Treat. 2017;165: 193–200. doi: 10.1007/s10549-017-4325-2 28578505 PMC5511313

[14] Hall JM, Lee MK, Newman B, Morrow JE, Anderson LA, Huey B, et al. Linkage of early-onset familial breast cancer to chromosome 17q21. Science. 1990;250: 1684–1689. doi: 10.1126/science.2270482 2270482

[15] Wooster R, Neuhausen SL, Mangion J, Quirk Y, Ford D, Collins N, et al. Localization of a breast cancer susceptibility gene, BRCA2, to chromosome 13q12–13. Science. 1994;265: 2088–2090. doi: 10.1126/science.8091231 8091231

[16] Ford D, Easton DF, Stratton M, Narod S, Goldgar D, Devilee P, et al. Genetic heterogeneity and penetrance analysis of the BRCA1 and BRCA2 genes in breast cancer families. The Breast Cancer Linkage Consortium. Am J Hum Genet. 1998; 62: 676–689. doi: 10.1086/301749 9497246 PMC1376944

[17] Couch FJ, Nathanson KL, Offit K. Two decades after BRCA: Setting paradigms in personalized cancer care and prevention. Science. 2014;343: 1466–1470. doi: 10.1126/science.1251827 24675953 PMC4074902

[18] Kuchenbaecker KB, Hopper JL, Barnes DR, Phillips KA, Mooij TM, Roos-Blom MJ, et al. Risks of breast, ovarian, and contralateral breast cancer for BRCA1 and BRCA2 mutation carriers. JAMA. 2017;317: 2402. doi: 10.1001/jama.2017.7112 28632866

[19] Winters S, Martin C, Murphy D, Shokar NK. Breast cancer epidemiology, prevention, and screening. Prog Mol Biol Transl Sci. 2017;151: 1–32. doi: 10.1016/bs.pmbts.2017.07.002 29096890

[20] Lord CJ, Ashworth A. PARP inhibitors: The first synthetic lethal targeted therapy. Science. 2017;355: 1152–8. 28302823 10.1126/science.aam7344 PMC6175050

[21] Litton JK, Rugo HS, Ettl J, Hurvitz SA, Gonçalves A, Lee KH. et al. Talazoparib in patients with advanced breast cancer and a germline BRCA mutation. N Engl J Med. 2018;379: 753–763. doi: 10.1056/NEJMoa1802905 30110579 PMC10600918

[22] Robson M, Im SA, Senkus E, Xu B, Domchek SM, Masuda N, et al. Olaparib for metastatic breast cancer in patients with a germline BRCA mutation. N Engl J Med. 2017;377: 523–533. doi: 10.1056/NEJMoa1706450 28578601

[23] Tutt AN, Garber JE, Kaufman B, Viale G, Fumagalli D, Rastogi P, et al. Adjuvant Olaparib for patients with BRCA1- or BRCA2-mutated breast cancer. N Engl J Med. 2021;384: 2394–2405. doi: 10.1056/NEJMoa2105215 34081848 PMC9126186

[24] Su Y, Yao Q, Xu Y, Yu C, Zhang J, Wang Q, et al. Characteristics of germline non-BRCA mutation status of high-risk breast cancer patients in China and correlation with high-risk factors and multigene testing suggestions. Front Genet. 2021;12: 674094. doi: 10.3389/fgene.2021.674094 34917121 PMC8670232

[25] Coppa A, Nicolussi A, D’Inzeo S, Capalbo C, Belardinilli F, Colicchia V, et al. Optimizing the identification of risk-relevant mutations by multigene panel testing in selected hereditary breast/ovarian cancer families. Cancer Med. 2018;7: 46–55. doi: 10.1002/cam4.1251 29271107 PMC5773970

[26] Metcalfe KA, Finch A, Poll A, Horsman D, Kim-Sing C, Scott J, et al. Breast cancer risks in women with a family history of breast or ovarian cancer who have tested negative for a BRCA1 or BRCA2 mutation. Br J Cancer. 2009;100: 421–425. doi: 10.1038/sj.bjc.6604830 19088722 PMC2634722

[27] Shiovitz S, Korde LA. Genetics of breast cancer: a topic in evolution. Ann Oncol. 2015;26: 1291–1299. doi: 10.1093/annonc/mdv022 25605744 PMC4478970

[28] Nielsen FC, Hansen TV, Sørensen CS. Hereditary breast and ovarian cancer: New genes in confined pathways. Nat Rev Cancer. 2016;16: 599–612. doi: 10.1038/nrc.2016.72 27515922

[29] Daly MB, Pal T, Berry MP, Buys SS, Dickson P, Domchek SM, et al. Genetic/familial high-risk assessment: Breast, ovarian, and pancreatic, Version 2.2021, NCCN Clinical Practice Guidelines in Oncology. J Natl Compr Canc Netw 2021;19: 77–102. doi: 10.6004/jnccn.2021.0001 33406487

[30] Colas C, Golmard L, de Pauw A, Caputo SM, Stoppa-Lyonnet D. Decoding hereditary breast cancer” benefits and questions from multigene panel testing. The Breast. 2019;45: 29–35. doi: 10.1016/j.breast.2019.01.002 30822622

[31] Yang X, Wu J, Lu J, Liu G, Di G, Chen C, et al. Identification of a comprehensive spectrum of genetic factors for hereditary breast cancer in a Chinese population by next-generation sequencing. PLoS One. 2015;10: e0125571. doi: 10.1371/journal.pone.0125571 25927356 PMC4415911

[32] Shin HC, Lee HB, Yoo TK, Lee ES, Kim RN, Park B, et al. Detection of germline mutations in breast cancer patients with clinical features of hereditary cancer syndrome using a multi-gene panel test. Cancer Res Treat. 2020;52(3): 697–713. doi: 10.4143/crt.2019.559 32019277 PMC7373875

[33] Nassar A, Zekri AR, Kamel MM, Elberry MH, Lotfy MM, Seadawy MG, et al. Frequency of pathogenic germline mutations in early and late onset familial breast cancer patients using multi-gene panel sequencing: An Egyptian study. Genes. 2022;14: 106. doi: 10.3390/genes14010106 36672847 PMC9858960

[34] Al Hannan F, Keogh MB, Taha S, Al Buainain L. Characterization of BRCA1 and BRCA2 genetic variants in a cohort of Bahraini breast cancer patients using next-generation sequencing. Mol Genet Genom Med. 2019;7: e00771. 10.1002/mgg3.771 PMC6625152 31131559

[35] Li MM, Datto M, Duncavage EJ, Kulkarni S, Lindeman NI, Roy S, et al. Standards and guidelines for the interpretation and reporting of sequence variants in cancer. A joint consensus recommendation of the Association for Molecular Pathology, American Society of Clinical Oncology, and College of American Pathologists. J Mol Diagn. 2017;19(1): 4–23. 27993330 10.1016/j.jmoldx.2016.10.002 PMC5707196

[36] Li Q, Wang K. InterVar: clinical interpretation of genetic variants by the 2015 ACMG-AMP guidelines. Am J Hum Genet. 2017;100: 267–280. doi: 10.1016/j.ajhg.2017.01.004 28132688 PMC5294755

[37] Wang D, Li J, Wang Y, Wang E. A comparison on predicting functional impact of genomic variants. NAR Genom Bioiform. 2022;4(1): 122. doi: 10.1093/nargab/lqab122 35047814 PMC8759571

[38] Bernstein C, Prasad RA, Nfonsam V, Bernstei H. DNA damage, DNA repair and cancer. In new research directions in DNA repair; In Tech Open: London, UK, 2013.

[39] Sharbeen G, McCarroll J, Goldstein D, Phillips PA. Exploiting base excision repair to improve therapeutic approaches for pancreatic cancer. Front Nutr. 2015;2: 10. doi: 10.3389/fnut.2015.00010 25988138 PMC4428371

[40] Nguyen L, Martens JWM, Van Hoeck A, Cuppen E. Pan-cancer landscape of homologous recombination deficiency. Nat Commun. 2020;11: 5584. doi: 10.1038/s41467-020-19406-4 33149131 PMC7643118

[41] Findlay GM, Daza RM, Martin B, Zhang MD, Leith AP, Gasperini M, et al. Accurate functional classification of thousands of *BRCA1* variants with saturation genome editing. Nature. 2018;562: 217–222. 30209399 10.1038/s41586-018-0461-z PMC6181777

[42] Wu J, Lu LY, Yu X. The role of BRCA1 in DNA damage response. Protein Cell. 2010;1: 117–123. doi: 10.1007/s13238-010-0010-5 21203981 PMC3078634

[43] Hahnena E, Haukea J, Engelb C, Neidhardta G, Rhiema K, Schmutzlera RK. Germline mutations in triple-negative breast cancer. Breast Care. 2017;12: 15–19. doi: 10.1159/000455999 28611536 PMC5465748

[44] Turk A. PARP Inhibition in BRCA-Mutant Breast Cancer. Cancer. 2018;124(12): 2498–2506. 29660759 10.1002/cncr.31307 PMC5990439

[45] Barchiesi G, Roberto M, Verrico M, Vici P, Tomao S, Tomao F. Emerging role of PARP inhibitors in metastatic triple negative breast cancer. Current scenario and future perspectives. Front Oncol. 2021;11: 769280. doi: 10.3389/fonc.2021.769280 34900718 PMC8655309

[46] Liu X, Wu K, Zheng D, Luo C, Fan Y, Zhong X, et al. Efficacy and safety of PARP inhibitors in advanced or metastatic triple-negative breast cancer: A systematic review and meta-analysis. Front Oncol. 2021;11: 742139. doi: 10.3389/fonc.2021.742139 34778059 PMC8581463

[47] Armstrong N, Ryder S, Forbes C, Ross J, Quek RGW. A systematic review of the international prevalence of BRCA mutation in breast cancer. Clin Epidemiol. 2019;11: 543–561. doi: 10.2147/CLEP.S206949 31372057 PMC6628947

[48] Bora E, Caglayan AO, Koc A, Cankaya T, Ozkalayci H, Kocabey M, et al. Evaluation of hereditary/familial breast cancer patients with multigene targeted next generation sequencing panel and MLPA analysis in Turkey. Cancer Genet. 2022;262: 118–133. doi: 10.1016/j.cancergen.2022.02.006 35220195

[49] Kwong A, Shin VY, Chen J, Cheuk IWY, Ho CYS, Au CH, et al. Germline mutation in 1338 BRCA-negative Chinese hereditary breast and/or ovarian cancer patients. J Mol Diagn. 2020;22(4): 544–554. 32068069 10.1016/j.jmoldx.2020.01.013

[50] Jalkh N, Chouery E, Haidar Z, Khater C, Atallah D, Ali H, et al. Next-generation sequencing in familial breast cancer patients from Lebanon. BMC Med Genom. 2017;10: 8. doi: 10.1186/s12920-017-0244-7 28202063 PMC5312584

[51] Bu R, Siraj AK, Al-Obaisi KA, Beg S, Al Hazmi M, Ajarim D, et al. Identification of novel BRCA founder mutations in Middle Eastern Breast cancer patients using capture and Sanger sequencing analysis. Int J Cancer. 2016;139: 1091–1097. doi: 10.1002/ijc.30143 27082205 PMC5111783

[52] AL Bader SM, Bugrein HA, Al-Sulaiman RJ. Genotype and phenotype correlation of breast cancer in BRCA mutation carriers and non-carriers. J Cancer Sci Ther. 2017;9: 358–364.

[53] Kairupan C, Scott RJ. Base excision repair and the role of MUTYH. Hered Cancer Clin Pract. 2007;5: 199–209. doi: 10.1186/1897-4287-5-4-199 19725997 PMC2736980

[54] Curia MC, Catalano T, Aceto GM. MUTYH: Not just polyposis. World J Clin Oncol. 2020;11: 428–49. doi: 10.5306/wjco.v11.i7.428 32821650 PMC7407923

[55] Sieber OM, Lipton L, Crabtree M, Heinimann K, Fidalgo P, Phillips RK, et al. Multiple colorectal adenomas, classic adenomatous polyposis, and germ-line mutations in MYH. N Engl J Med. 2003;348: 791. doi: 10.1056/NEJMoa025283 12606733

[56] Beiner ME, Zhang WW, Zhang S, Gallinger S, Sun P, Narod SA. Mutations of the MYH gene do not substantially contribute to the risk of breast cancer. Breast Cancer Res Treat. 2009;114: 575–8. doi: 10.1007/s10549-008-0042-1 18454351

[57] Fulk K, LaDuca H, Black MH, Qian D, Tina Y, Yussuf A, et al. Monoallelic MUTYH carrier status is not associated with increased breast cancer risk in a multigene panel cohort. Fam Cancer. 2019;18: 197–201. doi: 10.1007/s10689-018-00114-4 30582135

[58] Rennert G, Lejbkowicz F, Cohen I, Pinchev M, Rennert HS, Barnett-Griness O. MutYH mutation carriers have increased breast cancer risk. Cancer. 2012;118: 1989–93. doi: 10.1002/cncr.26506 21952991

[59] Wasielewski M, Out AA, Vermeulen J, Nielsen M, Van Den Ouweland A, Tops CMJ, et al. Increased MUTYH mutation frequency among Dutch families with breast cancer and colorectal cancer. Breast Cancer Res Treat. 2010;124: 635–41. doi: 10.1007/s10549-010-0801-7 20191381

[60] Lin PH, Kuo WH, Huang AC, Lu YS, Lin CH, Kuo SH, et al. Multiple gene sequencing for risk assessment in patients with early-onset or familial breast cancer. Oncotarget. 2016;7(7): 8310–20. doi: 10.18632/oncotarget.7027 26824983 PMC4884994

[61] Nunziato M, Di Maggio F, Pensabene M, Esposito MV, Starnone F, De Angelis C, et al. Multi-gene panel testing increases germline predisposing mutaions’ detection in a cohort of breast/ovarian cancer patients from Southern Italy. Front Med. 2022;9: 894358. 10.3389/fmed.2022.894358 PMC9403188 36035419

[62] Solano AR, Mele PG, Jalil FS, Liria NC, Podesta EJ, Gutiérrez LG. Study of the genetic variants in brca1/2 and non-brca genes in a population-based cohort of 2155 breast/ovary cancer patients, including 443 triple-negative breast cancer patients, in Argentina. Cancers. 2021;13: 2711. 34072659 10.3390/cancers13112711 PMC8198763

[63] Fonfria M, de Juan Jiménez I, Tena I, Chirivella I, Richart-Aznar P, Segura A, et al. Prevalence and clinicopathological characteristics of moderate and high- penetrance genes in non-BRCA1/2 breast cancer high-risk Spanish families. J Pers Med. 2021;11: 548. doi: 10.3390/jpm11060548 34204722 PMC8231620

[64] Cheadle JP, Sampson JR. MUTYH-associated polyposis—from defect in base excision repair to clinical genetic testing. DNA Repair (Amst). 2007;6: 274–9. doi: 10.1016/j.dnarep.2006.11.001 17161978

[65] Jiricny J. The multifaceted mismatch-repair system. Nat Rev Mol Cell Biol. 2006;7: 335–346. doi: 10.1038/nrm1907 16612326

[66] Pecina-Šlaus N, Kafka A, Salamon I, Bukovac A. Mismatch repair pathway, genome stability and cancer. Front Mol Biosci. 2020;7: 122. doi: 10.3389/fmolb.2020.00122 32671096 PMC7332687

[67] Win AK, Young JP, Lindor NM, Tucker KM, Ahnen DJ, Young GP, et al. Colorectal and other cancer risks for carriers and noncarriers from families with a DNA mismatch repair gene mutation: a prospective cohort study. J Clin Oncol. 2012;14: 958–964. doi: 10.1200/JCO.2011.39.5590 22331944 PMC3341109

[68] Smith CE, Mendillo ML, Bowen N, Hombauer H, Campbell CS, Desai A, et al. Dominant mutations in S. cerevisiae PMS1 identify the Mlh1‐Pms1 endonuclease active site and an exonuclease 1‐independent mismatch repair pathway. PLoS Genet. 2013;9(10): e1003869. doi: 10.1371/journal.pgen.1003869 24204293 PMC3814310

[69] Peltomali P. Lynch syndrome genes. Familial Cancer. 2005;4: 227–232. doi: 10.1007/s10689-004-7993-0 16136382

[70] Koivuluoma S, Winqvist R, Keski-Filppula R, Kuismin O, Moilanen J, Pylkäs K. Evaluating the role of MLH3 p.Ser1188Ter variant in inherited breast cancer predisposition. Genet Med. 2020;22(3): 663–664. doi: 10.1038/s41436-019-0694-8 31686011 PMC7056660

[71] Shao D, Cheng S, Guo F, Zhu C, Yuan Y, Hu K, et al. Prevalence of hereditary breast and ovarian cancer (HBOC) predisposition gene mutations among 882 HBOC high-risk Chinese individuals. Cancer Sci. 2020;111: 647–657. doi: 10.1111/cas.14242 31742824 PMC7004523

[72] Cavaillé M, Uhrhammer N, Privat M, Ponelle-Chachuat F, Gay-Bellile M, Lepage M, et al. Analysis of 11 candidate genes in 849 adult patients with suspected hereditary cancer predisposition. Genes Chromosomes Cancer. 2021;60: 73–78. doi: 10.1002/gcc.22911 33099839 PMC7756731

[73] Hu L, Sun J, Li Z, Qu Z, Liu Y, Wan Q, et al. Clinical relevance of pathogenic germline variants in mismatch repair genes in Chinese breast cancer patients. NPJ Breast Cancer. 2022;8: 52. doi: 10.1038/s41523-022-00417-x 35449176 PMC9023502

[74] AlHarbi M, Mobark NA, AlJabarat WAR, ElBardis H, AlSolme E, Hamdan AB, et al. Investigating the prevalence of pathogenic variants in Saudi Arabian patients with familial cancer using a multigene next generation sequencing panel. Oncotarget. 2023;14: 580–594. doi: 10.18632/oncotarget.28457 37306523 PMC10259259

[75] Alanazi M, Parine NR, Shaik JP, Al Naeem A, Aldhaian S. Targeted sequencing of crucial cancer causing genes of breast cancer in Saudi patients. Saudi J Biol Sci. 2020;27: 2651–2659. doi: 10.1016/j.sjbs.2020.05.047 32994724 PMC7499116

[76] Amemiya Y, Bacopulos S, Al-Shawarby M, Al-Tamimi D, Naser W, Ahmed A, et al. A comparative analysis of breast and ovarian cancer-related gene mutations in Canadian and Saudi Arabian patients with breast cancer. Anticancer Res. 2015;35: 2601–2610. 25964535

[77] Altinoz A, Al Ameri M, Qureshi W, Boush N, Nair SC, Abdel-Aziz A. Clinicopathological characteristics of gene-positive breast cancer in the United Arab Emirates. The Breast 2020;53: 119e124. doi: 10.1016/j.breast.2020.07.005 32745951 PMC7398969

[78] Willoughby A, Andreassen PR, Toland AE. Genetic testing to guide risk-stratified screens for breast cancer. J Pers Med. 2019;9: 15. doi: 10.3390/jpm9010015 30832243 PMC6462925

